# Homogeneity and tolerance to heat of monolayer MoS_2_ on SiO_2_ and h-BN†

**DOI:** 10.1039/c8ra01849a

**Published:** 2018-04-06

**Authors:** Ho-Jong Kim, Daehee Kim, Suyong Jung, Myung-Ho Bae, Sam Nyung Yi, Kenji Watanabe, Takashi Taniguchi, Soo Kyung Chang, Dong Han Ha

**Affiliations:** Quantum Technology Institute, Korea Research Institute of Standards and Science Daejeon 34113 Republic of Korea dhha@kriss.re.kr; Department of Electronic Material Engineering, Korea Maritime and Ocean University Busan 49112 Republic of Korea; National Institute for Materials Science 1-1 Namiki Tsukuba 305-0044 Japan; Department of Physics, Yonsei University Seoul 03722 Republic of Korea

## Abstract

We investigated the homogeneity and tolerance to heat of monolayer MoS_2_ using photoluminescence (PL) spectroscopy. For MoS_2_ on SiO_2_, the PL spectra of the basal plane differ from those of the edge, but MoS_2_ on hexagonal boron nitride (h-BN) was electron-depleted with a homogeneous PL spectra over the entire area. Annealing at 450 °C rendered MoS_2_ on SiO_2_ homogeneously electron-depleted over the entire area by creating numerous defects; moreover, annealing at 550 °C and subsequent laser irradiation on the MoS_2_ monolayer caused a loss of its inherent crystal structure. On the other hand, monolayer MoS_2_ on h-BN was preserved up to 550 °C with its PL spectra not much changed compared with MoS_2_ on SiO_2_. We performed an experiment to qualitatively compare the binding energies between various layers, and discuss the tolerance of monolayer MoS_2_ to heat on the basis of interlayer/interfacial binding energy.

## Abstract 2

We investigated the homogeneity and tolerance to heat of monolayer MoS_2_ using photoluminescence (PL) spectroscopy.

## Graphical abstract

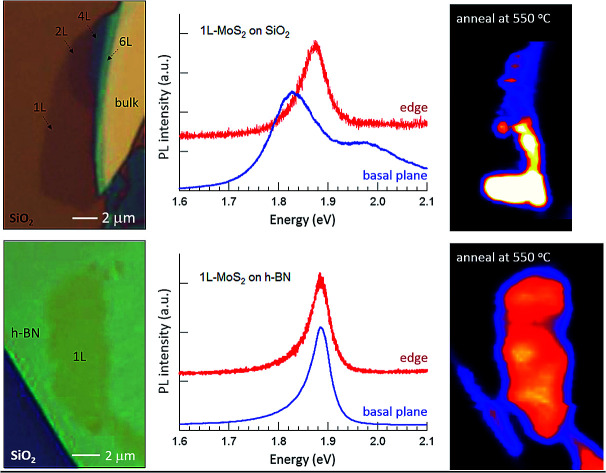

## Introduction

Transition metal dichalcogenides (TMDCs), such as molybdenum disulfide (MoS_2_), molybdenum diselenide (MoSe_2_), tungsten disulfide (WS_2_), and tungsten diselenide (WSe_2_), are two-dimensional (2D) semiconducting materials with strong photoluminescence (PL) emission in the visible or near-infrared spectral regions, which makes them attractive in the development of electronic and optoelectronic devices.^1–3^ TMDC monolayers exfoliated on various substrates are doped with electrons, or holes, induced by the charged impurities trapped at the TMDC-substrate interfaces. As the electrons/holes are depleted, PL emissions from TMDC monolayers are greatly enhanced in intensity and shift toward higher energies by the transformation of trions (charged excitons) into excitons.^1,4–6^ Thus, PL emission has been used to analyze the local charge density, defects, and strain on TMDC monolayers in combination with the results of Raman spectroscopy.^7–13^

Properties of 2D materials depend on the number of layers, defects, substrate, and so on. Monolayer MoS_2_ is a direct bandgap semiconductor with strong PL emission at 1.8–1.9 eV, while bulk MoS_2_ has an indirect bandgap at an energy approximately 0.6 eV lower than the monolayer.^14,15^ Photoluminescence maps of monolayer MoS_2_ on SiO_2_ have shown that the PL peak energy of the edges might differ from that of the crystalline basal plane (interior),^16^ and moreover, thermal annealing at 450 °C or higher creates defects on its basal plane with a large enhancement of PL emission.^1,7^ It has been reported that the PL/Raman properties as well as carrier mobility of MoS_2_ are affected by the substrate because of the changes in the doping level, extrinsic charge trap density, and optical interference within the substrate.^17–20^ In the present work, we went further to investigate the homogeneity of doping and tolerance to heat of monolayer MoS_2_ on substrates SiO_2_ and hexagonal boron nitride (h-BN) using PL spectroscopy. We found that monolayer MoS_2_ on SiO_2_ decomposed and lost its inherent crystal structure during thermal annealing up to 550 °C and subsequent optical mapping processes, while monolayer MoS_2_ on h-BN, having a homogenous PL spectra over the entire area including both basal plane and edge, was well preserved. Our results are expected to be useful in the development of nano-devices that require homogeneous 2D materials and reliable functionality under harsh environmental conditions, such as high temperatures.

## Experimental

MoS_2_ and h-BN flakes were prepared on Si substrates capped with 300 nm-thick SiO_2_ by mechanically exfoliating MoS_2_ (SPI Supplies) and h-BN crystals (National Institute for Materials Science) onto the substrates. MoS_2_ flakes were also prepared on SiO_2_/Si substrates coated with water-soluble poly-styrenesulfonic (PSS) and poly(methyl methacrylate) (PMMA). MoS_2_ monolayers were identified by optical microscopy and confirmed by Raman spectroscopy. For the fabrication of MoS_2_ and h-BN vertical heterostructures, MoS_2_ monolayers on the PMMA layer, obtained by removing the PSS layer, were brought into contact with thick h-BN flakes previously prepared on SiO_2_ using a micro-manipulator, followed by removal of the PMMA layer with acetone.^21^ The detailed fabrication process of the heterostructures is described in the ESI.† Samples were heat treated in a quartz tube (diameter = 90 mm) in a mixed atmosphere of Ar (1000 sccm) and H_2_ (100 sccm). Annealing was staged and accumulated: 1 hour at 250 °C, followed by 1 hour at 450 °C, and finally 1 hour at 550 °C. After each stage, the furnace was cooled down to room temperature.

Raman and PL spectra were obtained under ambient conditions in backscattering geometry using a laser line of 532 nm as exciting light. Scattered light was analysed using a Horiba Jobin Yvon LabRAM HR spectrometer equipped with a cooled charge-coupled device. A grating of 600 grooves per mm was used for the PL experiment, while a grating of 1800 grooves per mm was used for the Raman experiment. The exciting light was focused on the samples with a diameter (full width at half maximum: FWHM) of approximately 0.65 μm using a 100× objective lens, and the total laser power on the samples was fixed below 300 μW to prevent local heating or the deterioration of sample MoS_2_ by laser illumination.^9,22,23^ The Si Raman peak at 520.7 cm^−1^ was used as an internal reference to calibrate the Raman peaks of MoS_2_. In preliminary experiments, we monitored the PL and Raman spectra from an arbitrary point on monolayer MoS_2_ for 2 hours under our experimental conditions, with no meaningful changes observed. That is, the intensities and positions of the PL and Raman peaks did not change even when the laser light continuously illuminated the same point for 2 hours, thus confirming the absence of laser heating effects on the PL and Raman spectra. Except for the acquisition time, all other experimental parameters for the PL and Raman measurements were fixed, such as confocal hole size and neutral density filter. The thickness of the h-BN flakes was measured by tapping-mode atomic force microscopy.

## Results and discussion

**Fig. 1:**
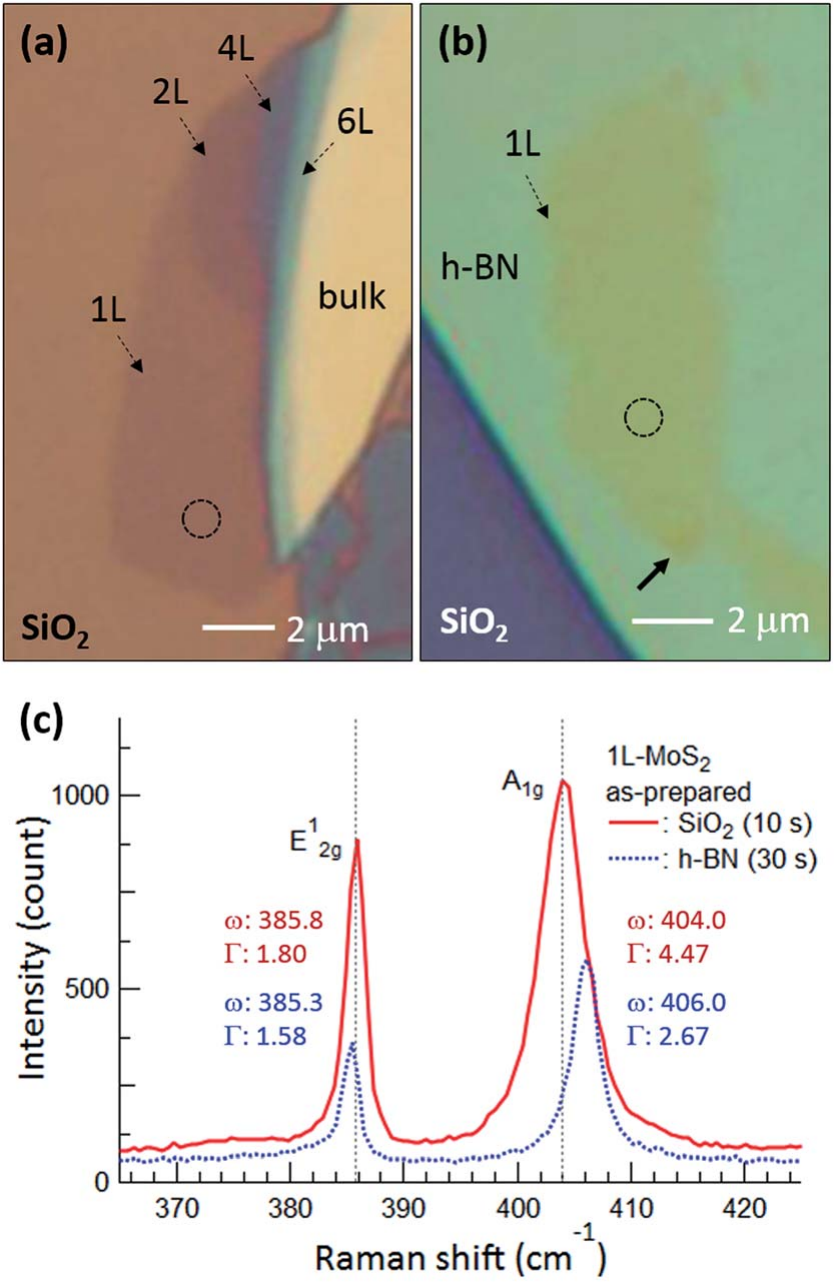
Optical microscopy images of MoS_2_ flakes on (a) SiO_2_ and (b) h-BN, where 1L, 2L, 4L, and 6L indicate monolayer, bilayer, quadlayer, and hexalayer, respectively, estimated using the position difference between Raman E_2g_^1^ and A_1g_ peaks.^24^ (c) Raman spectra of monolayer MoS_2_ obtained from the dotted circles in (a) and (b). Red and blue curves and numbers represent MoS_2_ on SiO_2_ and h-BN, respectively, and the times in parentheses indicate the acquisition time. *ω* and *Γ* are position and FWHM of the Raman peaks. Gray dotted lines in (c) denote the positions of the E_2g_^1^ and A_1g_ peaks of monolayer MoS_2_ on SiO_2_.

Fig. 1(a) and (b) are optical microscopy images of MoS_2_ flakes on SiO_2_ and h-BN, respectively. The lower left of the MoS_2_ flake on SiO_2_ is monolayer, with the rest of the flake consisting of multilayer or bulk MoS_2_. The MoS_2_ flake on 97 nm thick h-BN is entirely monolayer except for the bottom end indicated by the arrow, with tiny yellow flakes of MoS_2_ observed along the h-BN edge. Fig. 1(c) shows typical Raman spectra obtained from the basal plane of monolayer MoS_2_, where two Raman peaks were observed: an E_2g_^1^ peak (in-plane vibration) and an A_1g_ peak (out-of-plane vibration).^24–26^ Compared with the MoS_2_ on SiO_2_, we notice that both Raman peaks are shifted for MoS_2_ on h-BN. It has been reported that the E_2g_^1^ peak redshifts with increasing strain^25^ and likewise, an increase of the interlayer van der Waals force also redshifts the E_2g_^1^ peak due to stacking-induced structural changes or long-range coulombic interlayer interactions.^24,25,27^ On the other hand, the position and FWHM of the A_1g_ peak were affected predominantly by local electron density: the peak blueshifted and sharpened with electron depletion due to reduced electron–phonon interactions,^28^ although increased interlayer interactions could also lead to a blueshift of the A_1g_ peak by increasing the restoring force.^26^ The FWHM of the A_1g_ peak was 4.47 cm^−1^ on SiO_2_ but decreased to 2.67 cm^−1^ on h-BN. The Raman spectra indicate that monolayer MoS_2_ on h-BN was electron depleted compared with that on SiO_2_ and/or that the monolayer MoS_2_ attached more strongly to the h-BN than to the SiO_2_, as discussed in detail later.

**Fig. 2:**
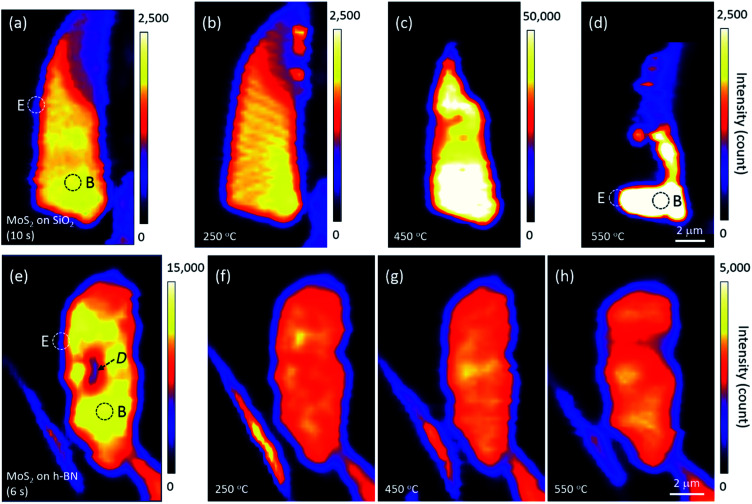
Changes in the PL intensity maps of MoS_2_ flakes on (a–d) SiO_2_ and (e–h) h-BN upon annealing. Acquisition times for each measurement are denoted in parentheses. The mapping step was 0.25 μm, Dotted circles denote the typical edge (“E”) and basal plane (“B”) of monolayer MoS_2_, and the dark area *D* in (e) is believed to be organic residue from the fabrication process that disappeared with annealing.

Annealing effects on the PL peak intensity (amplitude) maps of MoS_2_ flakes are shown in Fig. 2. PL intensities significantly decreased with increasing thickness such that those of 6L and bulk MoS_2_ appeared dark on the maps of Fig. 2(a)–(d). We noticed that the topography of the maps changed with each annealing, suggesting that the distribution of local charge density might be changed by annealing. A PL signal from MoS_2_ was also observed along the edge of the thick h-BN where tiny flakes of MoS_2_ were scattered.

The PL intensity of monolayer MoS_2_ on SiO_2_ was enhanced by approximately 20 times after annealing at 450 °C (see the scale bars next to the maps in Fig. 2). While the PL intensity of bilayer was slightly enhanced, those of multilayer and bulk were not changed on the whole, although they appear dark in Fig. 2(c). However, the PL intensity of monolayer MoS_2_ decreased significantly by annealing at 550 °C, with a large part disappearing from the PL map as shown in Fig. 2(d), indicating that monolayer MoS_2_ on SiO_2_ could no longer retain its intrinsic hexagonal crystal structure. Optical microscopy images showed that a part of the bilayer MoS_2_ on SiO_2_ was also decomposed, whereas thicker or multilayer MoS_2_ was well preserved. Our results for MoS_2_ on SiO_2_ are consistent with the work of Liu and coworkers on oxygen etching activity with various numbers of graphene layers on SiO_2_, which was discussed on the basis of substrate-induced deformations and preexisting defects;^29^ they showed that oxidative etching proceeded faster in single layers than in multi-layers during annealing in an O_2_/Ar gas flow, and moreover, etching did not occur on the defect-free basal plane of triple or thicker layer graphene at or below 600 °C.

Many experimental results have shown that defects can be created in monolayer MoS_2_ on SiO_2_*via* thermal annealing, plasma treatment, electron/ion irradiation, or laser irradiation.^1,2,7,8,30^ Moreover, high-resolution transmission electron microscopy images have verified S-vacancies—the most common type of defect—in electron-irradiated monolayer MoS_2_.^31^ Tongay, Nan, and their coworkers also reported that thermal annealing at 450 °C or higher creates S-vacancies by breaking the S–Mo–S bonds of MoS_2_.^1,7^ Annealing at 550 °C removes even more S atoms, such that monolayer MoS_2_ is considered to be fragmented into nano-domains that can be easily decomposed by laser light; this is in contrast to thicker or multilayer MoS_2_ in which all atoms are bonded horizontally and vertically. Here, Fig. 2 suggests that the interfacial interaction between MoS_2_ and SiO_2_ is weaker than the interlayer interaction between layers of MoS_2_ because of impurities at the MoS_2_–SiO_2_ interface and SiO_2_ surface roughness.^19–21^ Previous results on the comparison of binding energies between the interlayer/interface support our results. The interlayer binding energy between MoS_2_ layers calculated using advanced density-functional theory was 330 mJ m^−2^, and the energy required to peel off a single layer from the surface of a multilayer structure increased with the number of layers.^32^ On the other hand, the interfacial binding energy of MoS_2_ on SiO_2_ substrates measured by Deng and coworkers was 170 mJ m^−2^.^33^

We argue that the tolerance of monolayer MoS_2_ to heat is influenced by the interlayer/interfacial binding energy as well as the interatomic bonding forces in the layer. In fact, monolayer MoS_2_ on h-BN was well preserved even after annealing at 550 °C and subsequent PL mapping. Fig. 2 strongly indicates that due to the atomically flat, impurity-free surface of newly exfoliated h-BN,^19,21^ interfacial binding between MoS_2_ and h-BN is stronger than that between MoS_2_ and SiO_2_, which is consistent with the results of the Raman experiments shown in Fig. 1(c). Our results are in good agreement with the dependence of MoS_2_ interfacial binding energy on substrate roughness as measured by Deng and coworkers.^33^ Strong interfacial binding is considered to enhance the tolerance of monolayer MoS_2_ on h-BN to heat.

**Fig. 3:**
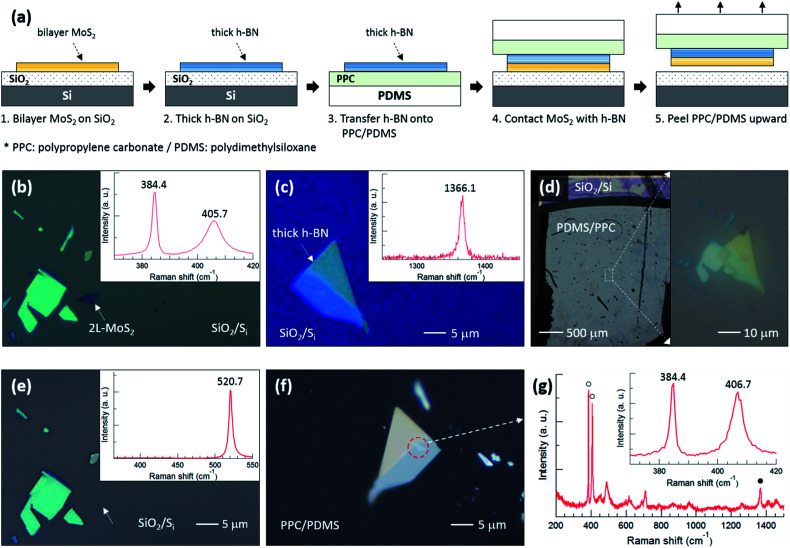
(a) Schematic diagram of the experimental process to qualitatively compare the binding energies between various layers. (b and c) Optical microscopy images with corresponding Raman spectra for steps 1 and 2 in (a), respectively. (d) Optical microscopy image for steps 3 and 4 in (a). The right image is an enlargement of the white rectangular region on the left. (e and f) Optical microscopy images for the lower and upper sections of step 5 in (a), respectively, with corresponding Raman spectrum in (e). (g) Raman spectrum obtained from the red dotted circle in (f).

To support our argument, we performed an experiment to qualitatively compare the binding energies between the various layers as summarized in Fig. 3. The flake designated by a white dotted arrow in Fig. 3(b) is bilayer MoS_2_, the thickness of which was determined using the position difference between the E_2g_^1^ and A_1g_ peaks in the inset Raman spectrum. The h-BN flake in Fig. 3(c) was also confirmed using the Raman spectrum. Fig. 3(d) shows optical images of PDMS/PPC/h-BN placed on MoS_2_/SiO_2_/Si; the right image demonstrates that the thick h-BN is precisely positioned on the bilayer MoS_2_. Fig. 3(e) shows that the bilayer MoS_2_ was completely separated from SiO_2_, as only a single Si Raman peak at 520.7 cm^−1^ was observed from the position where bilayer MoS_2_ was located. The bilayer MoS_2_ flake was then transferred onto the thick h-BN, as can be observed in the red dashed circle in Fig. 3(f), after peeling PDMS/PPC upward. In the Raman spectrum of Fig. 3(g) obtained from the red dashed circle, open (closed) circles denote Raman peaks of bilayer MoS_2_ (h-BN), while other peaks are from the PDMS/PPC. The inset in Fig. 3(g) shows the Raman spectrum of the MoS_2_ flake in detail. The A_1g_ peak at higher frequency was blueshifted but the position of the E_2g_^1^ peak at lower frequency was unchanged compared with those of the MoS_2_ flake on SiO_2_, indicating a depletion of electrons from MoS_2_ on h-BN compared with that on SiO_2_. The position difference between the E_2g_^1^ and A_1g_ peaks verifies that it is bilayer—not monolayer—MoS_2_. That is, bilayer MoS_2_ was not separated between the layers of MoS_2_ nor at the interface between MoS_2_ and h-BN, but rather was separated at the interface between MoS_2_ and SiO_2_. Our results demonstrate that the MoS_2_–SiO_2_ interfacial binding energy is weaker than the MoS_2_–MoS_2_ interlayer binding energy as well as the MoS_2_–h-BN interfacial binding energy.

**Fig. 4:**
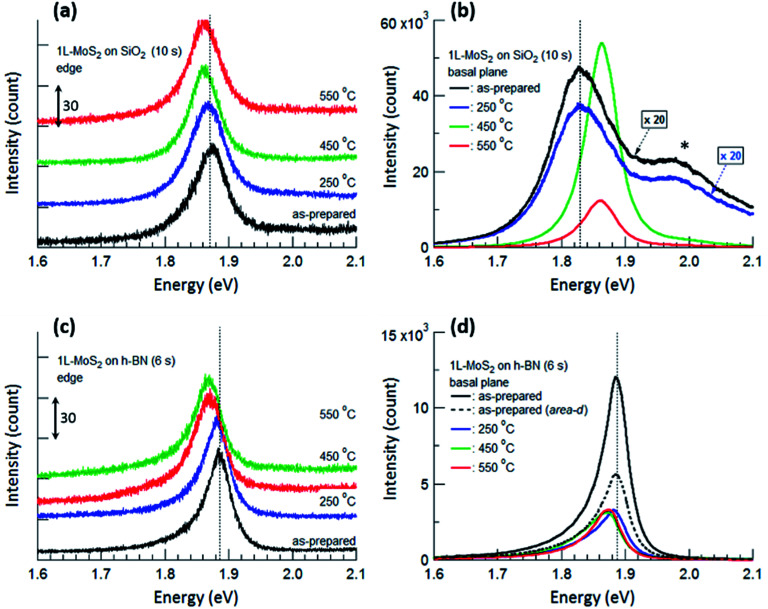
PL spectra obtained from the edge (a and c) and basal plane (b and d) of monolayer MoS_2_ shown in Fig. 2. The PL spectra of as-prepared MoS_2_ and MoS_2_ annealed at 250 °C are multiplied by 20 in (b). The shoulder peak at 1.98 eV marked by an asterisk in (b) is due to the splitting of the monolayer MoS_2_ valence band. PL spectra obtained from the edges are shifted vertically for ease of viewing, and the unit value of the *Y*-axis in (a) and (c) is 30 count. The times in parentheses denote the acquisition time for each measurement. Vertical dotted lines denote the PL peak positions of the as-prepared MoS_2_ monolayers.

Fig. 4 displays PL spectra obtained from the typical edge and basal plane of monolayer MoS_2_ on SiO_2_ and h-BN. Compared with the PL spectrum of the as-prepared MoS_2_ on SiO_2_, that of MoS_2_ on h-BN greatly increased in intensity even though the acquisition time was reduced from 10 to 6 s; further, the PL peak due to valence band splitting disappeared.^6,14^ Moreover, the peak position shifted from 1.83 to 1.89 eV and the FWHM decreased from 0.11 to 0.04 eV on h-BN, verifying that MoS_2_ on h-BN was electron depleted compared with that on SiO_2_ due to the impurity-free surface of h-BN.^1,4,19^

The basal plane of the as-prepared monolayer MoS_2_ on SiO_2_ had a PL maximum of negatively charged trions at approximately 1.83 eV, whereas the edge had a single PL peak of excitons at approximately 1.87 eV. This can be ascribed to local electron depletion by foreign molecules adsorbed on the dangling bonds along the edge. The edge was defined as the points corresponding to the initial upturns of the PL intensity profiles across the edge of MoS_2_ (to be precise, the PL signal of the edge is the sum of the signals from the true edge and the basal plane near the edge since the laser light has a finite size). The difference in PL spectra between the basal plane and the edge of MoS_2_ on SiO_2_ was evident even after annealing at 250 °C. However, annealing at 450 °C resulted in remarkable changes in the PL spectrum of the basal plane *via* the chemisorption of foreign molecules, such as O_2_ and H_2_, at newly created defects; such changes include a shift of the peak position to approximately 1.86 eV, an increase in the peak intensity by more than 20 times, and the disappearance of the shoulder peak at 1.98 eV. On the other hand, the PL peak of the edge only slightly shifted toward a lower energy, such that both PL spectra of the basal plane and edge of MoS_2_ on SiO_2_ had a single peak at the same position of approximately 1.86 eV.

For monolayer MoS_2_ on h-BN, both PL peaks of the basal plane and edge of MoS_2_ unexpectedly had maxima at the same energy, as shown in Fig. 4(c) and (d), even though foreign molecules were considered to be chemisorbed on the edge. This tendency was not changed by thermal annealing up to 550 °C. Fig. 4 indicates that the PL properties and doping of electron-depleted MoS_2_ on h-BN is insensitive to the adsorption of electron-accepting molecules, which is contrary to the case of monolayer MoS_2_ on SiO_2_. In other words, electron transfer from the electron-depleted MoS_2_ to the adsorbed molecules is considered to be insignificant compared with that from MoS_2_ on SiO_2_, on which the electron transfer from an S-vacancy of MoS_2_ to a chemisorbed O_2_ molecule reaches 0.997 *e*.^7^ Our results show that with or without annealing, local electron density was nearly homogeneous over the entire monolayer MoS_2_ on h-BN, including both basal plane and edge.

Thermal annealing decreased the PL intensity of monolayer MoS_2_ on h-BN; in addition, the PL peaks of both the basal plane and edge of MoS_2_ shifted toward a lower energy. First-principle calculations showed that both O_2_ and H_2_ molecules physisorbed on monolayer MoS_2_ act as electron acceptors with charge transfer values of 0.04 *e* and 0.004 *e* for O_2_ and H_2_, respectively. In addition, the adsorption energies for O_2_ and H_2_ on ideal MoS_2_ were calculated to be −116 and −82 meV.^34^ Replacing some O_2_ molecules physisorbed on the basal plane of MoS_2_ with H_2_ molecules during annealing in a H_2_/Ar gas flow could increase the local electron density on the basal plane, resulting in the changes in the intensity and position of PL spectrum. Our results show that the electron-depleted MoS_2_ on h-BN responds to the substitution of O_2_ with H_2_ which results in the electron-doping, although it was insensitive to the adsorption of electron-accepting molecules. Nevertheless, we consider that few of the O_2_ molecules chemisorbed on the edge or other defects could be removed during thermal annealing since the binding energy of an O_2_ molecule on an S-vacancy of MoS_2_ calculated by first-principle method is −2.395 eV.^7^

## Conclusions

The PL spectra of the edge for the as-prepared MoS_2_ on SiO_2_ differed in peak position and shape from those of the basal plane, but annealing at 450 °C rendered the PL spectra homogeneous by creating many defects on the basal plane. On the other hand, MoS_2_ on h-BN demonstrated a homogenous PL spectra over the entire area regardless of annealing, although foreign molecules were expected to chemisorb to the dangling bonds along the edge. Electron-depleted MoS_2_ on h-BN responded to the adsorption of foreign molecules which results in the electron-doping, while it was insensitive to the adsorption of electron-accepting molecules. Monolayer MoS_2_ on SiO_2_ was further decomposed and almost lost its inherent crystal structure during thermal annealing at 550 °C and subsequent optical mapping process, while monolayer MoS_2_ was well preserved on h-BN. We contend that the tolerance of monolayer MoS_2_ to heat is influenced by interlayer/interfacial binding energy as well as the interatomic bonding forces in the layer, strongly supported by experimental results on the comparison of binding energies between various layers.

## Conflicts of interest

There are no conflicts to declare.

## Supplementary Material

RA-008-C8RA01849A-s001
