# Supplementary material for: Homogeneity and tolerance to heat of monolayer MoS2 on SiO2 and h-BN
Source: RSC Adv. 2018 Apr 6;8(23):12900–6. doi: 10.1039/c8ra01849a (PMC9079624; doi:10.1039/c8ra01849a)
Supplement: RA-008-C8RA01849A-s001 [file RA-008-C8RA01849A-s001.pdf]

Electronic Supplementary Material (ESI) for RSC Advances.
This journal is © The Royal Society of Chemistry 2018

Supplementary Information

Homogeneity and tolerance to heat of monolayer MoS_2 on SiO_2 and h-BN

Ho-Jong Kim,[a,d] Daehee Kim,[a] Suyong Jung,[a] Myung-Ho Bae,[a] Sam Nyung Yi,[b] Kenji Watanabe,[c] Takashi Taniguchi,[c] Soo Kyung Chang[d] and Dong Han Ha[*a]

[a]*Quantum Technology Institute, Korea Research Institute of Standards and Science, Daejeon 34113, Republic of Korea*

[b]*Department of Electronic Material Engineering, Korea Maritime and Ocean University, Busan 49112, Republic of Korea*

[c]*National Institute for Materials Science, 1-1 Namiki, Tsukuba 305-0044, Japan*

[d]*Department of Physics, Yonsei University, Seoul 03722, Republic of Korea*

*E-mail: dhha@kriss.re.kr

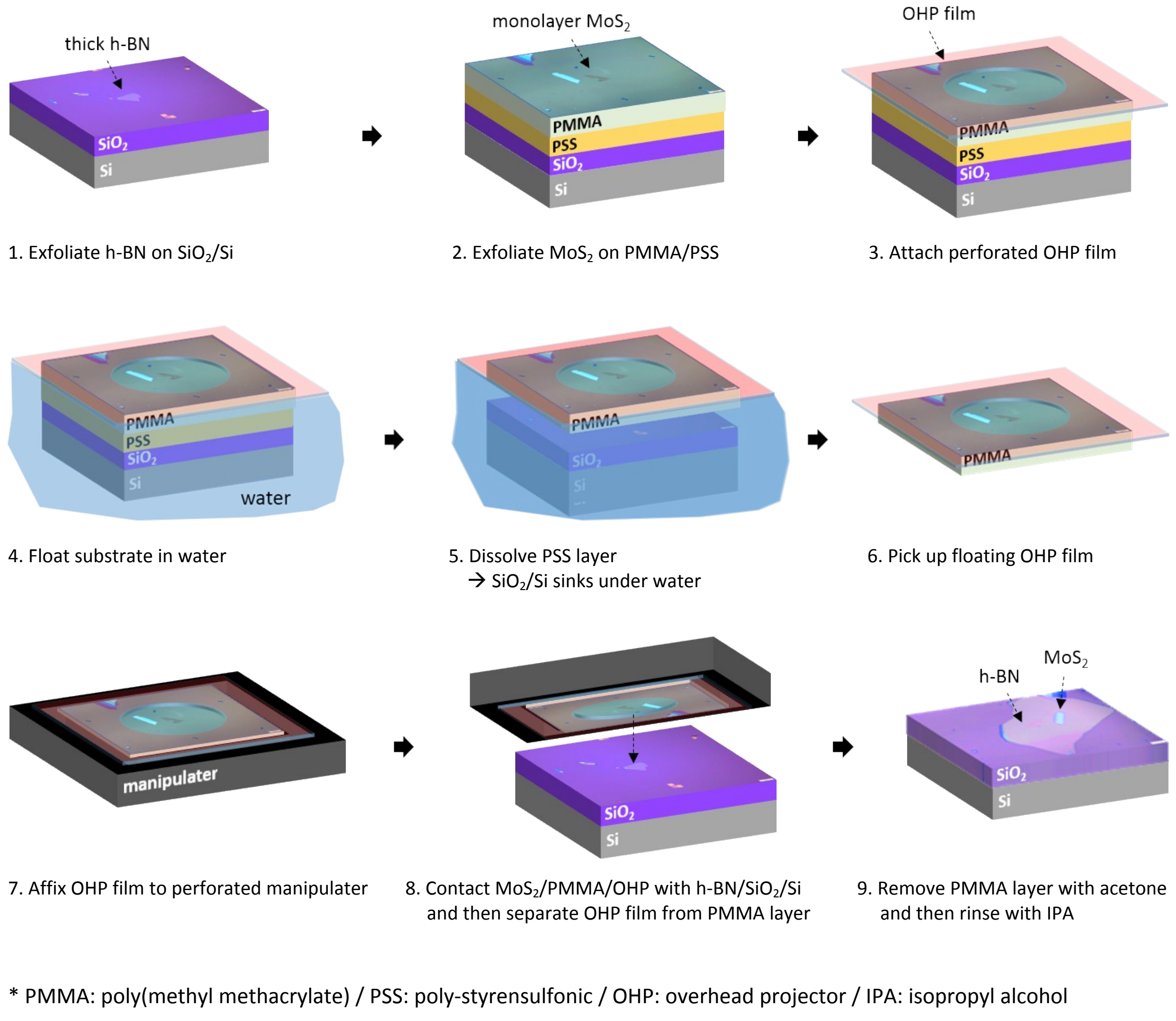

Fig. S1 Schematic diagram of the experimental process to fabricate MoS_2 and h-BN vertical heterostructures on SiO_2/Si substrate.
